# Fabrication of a Micro-Fluid Gathering Tool for the Gastrointestinal Juice Sampling Function of a Versatile Capsular Endoscope

**DOI:** 10.3390/s110706978

**Published:** 2011-07-04

**Authors:** Kyo-in Koo, Sangmin Lee, Dong-il Dan Cho

**Affiliations:** Inter-University Semiconductor Research Center and Automation System Research Institute, School of Electrical Engineering and Computer Science, Seoul National University, 599 Gwanak-ro Gwanak-gu, 151-744, Seoul, Korea; E-Mails: kkin76@snu.ac.kr (K.K.); sangmlee@snu.ac.kr (S.L.)

**Keywords:** micro-pump, Venturi tube, azobisisobutyronitrile, capsular endoscope

## Abstract

This paper presents a micro-fluid gathering tool for a versatile capsular endoscope that employs a solid chemical propellant, azobisisobutyronitrile (AIBN). The proposed tool consists of a micro-heater, an AIBN matrix, a Venturi tube, a reservoir, an inlet, and an outlet. The micro-heater heats the AIBN matrix to be decomposed into by-products and nitrogen gas. This nitrogen gas generates negative pressure passing through the Venturi tube. The generated negative pressure inhales a target fluid from around the inlet into the reservoir. All the parts are designed to be embedded inside a cylindrical shape with a diameter of 17 mm and a height of 2.3 mm in order to integrate it into a versatile developmental capsular endoscope without any scaledown. Two sets of the proposed tools are fabricated and tested: one is made of polydimethylsiloxane (PDMS) and the other is made of polymethylmethacrylate (PMMA). In performance comparisons, the PDMS gathering tool can withstand a stronger pulling force, and the PMMA gathering tool requires a less negative pressure for inhaling the same target fluid. Due to the instant and full activation of the thin AIBN matrix, both types of gathering tool show analogous performance in the sample gathering evaluation. The gathered volume is approximately 1.57 μL using approximately 25.4 μL of AIBN compound.

## 1. Introduction

Conventional gastrointestinal endoscopy is a widely used medical tool to assess the interior surface of the gastrointestinal tracts by inserting a tube through the throat. Most endoscopies not only provide gastrointestinal images, but can also take biopsies and perform simple surgical operations. In spite of these advantages, gastrointestinal endoscopies cannot reach the small intestines because of their inferior navigational function. During the endoscopic procedure, the thick tubes in patient’s throat produce continuous nausea in the patient. In addition, considerable training is required for expert endoscopy manipulation due to its manually controlled operating mechanism.

In order to improve theses disadvantages, Jacobsen developed endoradiosondes, also called radio-pills in 1957 [1]. Since Jacobsen’s pioneering development, significant research on a capsular endoscope has been performed during the last decade. Given Imaging (Yoqneam, Israel) has primarily commercialized a capsular endoscope, called M2A® in 2000 [2,3]. Following its work, Olympus (Tokyo, Japan) [4], Smart Pill Corporation (Buffalo, NY, USA) [5], VECTOR (Tübingen, Germany) [6], and Intelligent Microsystem Center (Seoul, Korea) [7] have presented various capsular endoscopes.

A capsular endoscope provides an attractive platform which can reach all the gastrointestinal tract from the esophagus to the duodenum with just only one swallowing. Until now, only an image transport module and a battery have been taken aboard this capsular platform. In order to substitute the conventional gastrointestinal endoscope, gastrointestinal juice sampling, navigation, biopsy, on-capsule diagnosis, drug injection, and simple surgery have been researched for a versatile capsular endoscope by collaborative research groups (VECTOR and Intelligent Microsystem Center) [6,7].

**Figure 1.**
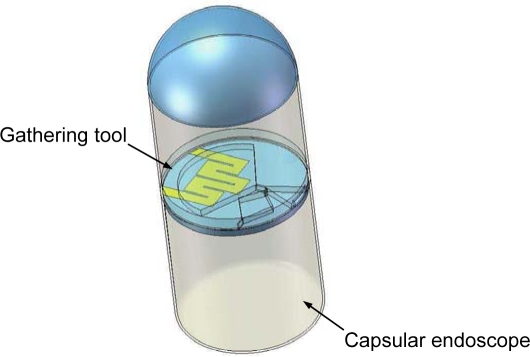
The conceptual schematic of a versatile capsular endoscope integrated with a gathering tool (In order to present clear concept, other components except for the gathering tool are omitted).

Figure 1 represents a conceptual schematic of a versatile capsular endoscope integrated with a gathering tool. In a gastro intestine the versatile capsular endoscope searches for gastrointestinal juice using a camera module, and then approaches the searched juice by a self-navigating tool. After approaching, a gathering tool harvests the target juice.

As a sub-group of the Intelligent Microsystem Center, our group has fabricated and studied a micro-fluid gathering tool for a gastrointestinal juice sampling function. Even though a plethora of micro-pumps have been reported [8], they cannot be utilized in a versatile capsular endoscope because of their device size, flow rate, and pumping volume. Especially, their practical device size which includes their fluidic power source (for example, syringe pump, compressed gas tank, and so on) is a primary hindrance for integration into a capsule.

The proposed gathering tool employs the compound azobisisobutyronitrile (AIBN) as a fluidic power source. The property of its heat-induced decomposition into nitrogen gas makes tens of millimeter scale possible as the practical device size. This device size enables integration into a developing versatile capsular endoscope without any scaledown. The proposed gathering tool is designed based on the Venturi tube principle. In a Venturi tube, the heat-decomposed nitrogen gas generates a negative pressure without requiring any micro-valve [9]. The generated negative pressure inhales a target fluid into a reservoir. In this research, two sets of the proposed tools are fabricated: one is made of polydimethylsiloxane (PDMS), and the other is made of polymethylmethacrylate (PMMA). Their performances are evaluated and compared to determine their medical application feasibility.

## 2. Concept and Design

**Figure 2.**
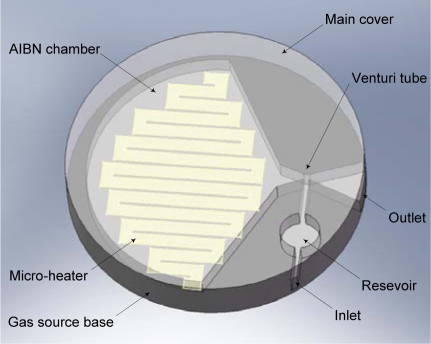
The conceptual schematic of a gathering tool (the AIBN matrix on the micro-heater is omitted for clarity).

The proposed tool is a bonded device with a gas source base component and a main cover component. The structure of the main cover contains an AIBN chamber, a Venturi-tube, a reservoir, an inlet, and an outlet. The gas source base has an AIBN matrix and a micro-heater on its surface. A conceptual schematic is shown in Figure 2. The micro-heater for the AIBN compound decomposition is aligned beneath the AIBN chamber. The AIBN chamber and the reservoir are joined into the outlet through the vena contracta section of the Venturi tube. When electric power heats the AIBN matrix by means of the micro-heater, the AIBN compound is decomposed liberating nitrogen gas. This nitrogen gas is accelerated at the vena contracta section of the Venturi tube. The accelerated gas generates negative pressure so that the negative pressure inhales a target fluid around the inlet, and then stores the inhaled fluid in the reservoir. The over-inhaled fluid is exhaled through the outlet with the accelerated nitrogen gas.

Some solid chemical propellants simply need thermal energy for gas generation so that they are widely applied in propulsion of micro-spacecraft in the space environment. However, the space applicable propellant materials work at high operating temperatures of over 400 °C. These materials release nitrogen gas with toxic by-products, such as CO, and C_x_H_y_, *etc.* [10]. However, AIBN compound decomposes at around 65–105 °C and produces inert nitrogen gas and a free radical (tetramethylsuccinonitrile, TMSN). This decomposition temperature is relatively low. In order to keep AIBN powder (Unisource, India) on the micro-heater under a shock or vibration environment, the AIBN powder is mixed with powdered Teflon® (Teflon® AF 2400, DuPont Korea, Korea) in a solvent solution (PF-5080, 3M Korea, Seoul, Korea) to be used as a solid matrix form [11]. In this research, the by-product material of the AIBN compound decomposition (TMSN) remains in the solid phase after the decomposition so that it remains in the solid Teflon® matrix. Therefore, it is expected not to affect the stored fluid in the reservoir during the gathering process. The low decomposition temperature and the solid by-product encourage the use of the AIBN matrix in this tool.

An Au/Ti thin film micro-heater was designed for the decomposition of the AIBN. In order to decompose the AIBN, a micro-heater should supply at least 105 °C considering the AIBN compound decomposition temperature of 65–105 °C [11–13]. The micro-heater is designed as a zigzag line with a length of 57.2 mm, a width of 1.4 mm, and a thickness of 750 Å/500 Å (Au/Ti).

**Figure 3.**
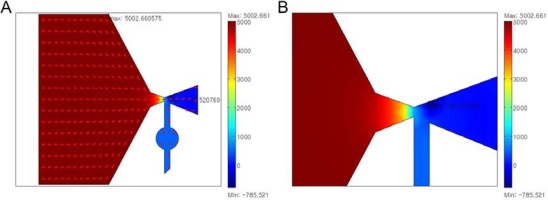
The simulation model and result of the gathering tool.

The design of the proposed tool is based on the Venturi tube principle. A simple two-dimensional Venturi tube channel was designed using the following equation, which originates from Bernoulli’s equation:

(1) $$VP=P_{1}-P_{2}=\frac{1}{2}\rho \nu_{1}^{2}\left(\frac{A_{1}^{2}}{A_{2}^{2}}-1\right)$$

where P_1_ and P_2_ are pressures, ρ is the density, v1 is fluid velocity, and A_1_ and A_2_ are channel areas. A simulation program, MULTIPHYSICSTM 3.2 (Comsol, Burlington, MA, USA), is used to design the Venturi tube dimensions. In this simulation, the governing equation is the Navier-Stokes equation. The fluid flow is assumed to be laminar flow. Figure 3 shows the simulation model and results. The AIBN chamber is simply represented by a rectangular shape to keep the calculations manageable. The widths of the vena contracta section and the inlet are set as 500 μm. The reservoir is a circular shape with an area of 3.14 mm^2^. When the incompressible fluid pressure around the AIBN chamber is 5 kPa, this model simulates that the generated negative pressure is 0.8 kPa.

## 3. Fabrication

**Figure 4.**
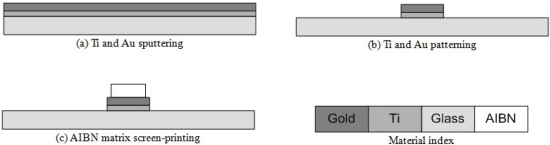
The fabrication process flow of the gas source base.

The gas source base and main cover are fabricated separately, and then bonded together. In this research, the main cover is made of PDMS and PMMA, respectively, for performance comparison. Figure 4 shows fabrication process flow of the gas source base. The micro-heater is fabricated by patterning of a 500 Å thick titanium film and a 750 Å thick gold film on a 500 μm glass wafer (Borofloat®, Schott, Mainz, Germany). The measured resistance of the fabricated micro-heater is 21.7 Ω. A glass wafer was chosen for the micro-heater substrate because of its lower thermal conductivity and higher electrical resistivity compared to the Si wafer [14]. The Ti film is used as an adhesion layer to improve adhesion between the glass wafer and the gold film. The pure AIBN powder is mixed with the Teflon® powder in a ratio of 5:1 in the PF-5080 solvent [11]. The AIBN matrix sol-gel is then screen-printed on the micro-heater, and cured at room temperature.

**Figure 5.**
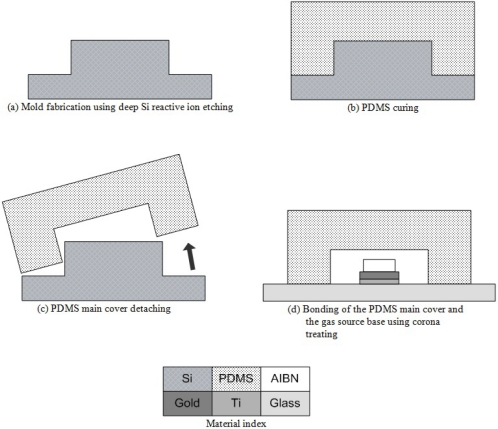
The fabrication process flow of the PDMS gathering tool.

In the case of the PDMS main cover, the soft lithography method is utilized. Figure 5 represents the soft lithography process flow and the PDMS bonding process flow for the PDMS gathering tool. The main cover design is patterned on a silicon wafer by photo lithography process and deep Si reactive ion etching process (500 μm etching). Gel state PDMS is poured on the patterned silicon mold, and then cured for 12 h on a 50 °C hotplate. The cured PDMS main cover is detached from the patterned Si mold. Wire electrode of a corona treater (BD-10AS, Electro-Technic Products, Inc., Chicago, IL, USA) passes back and forth approximately 1 cm above the bonding surface of the detached PDMS main cover and the gas source base. The treated surfaces are then contacted together and left on a 50 °C hotplate for 12 h. This bonding recipe is a modified version of Haubert’s recipe [15].

**Figure 6.**
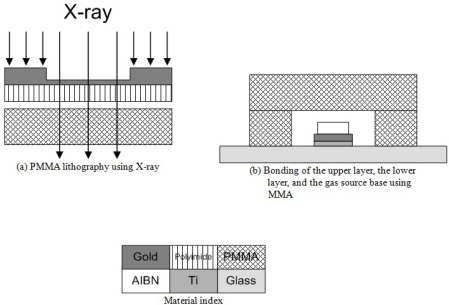
The fabrication process flow of the PMMA gathering tool.

The LiGA (Lithographie, Galvanoformung, Abformung; German acronym of Lithography, Electroplating, and Molding) process is utilized for the PMMA main cover. Figure 6 shows the LiGA process flow and PMMA boding process flow for the PMMA gathering tool. The soft lithography method fabricates the PDMS main cover as one piece of body at one step. However, the LiGA method cannot fabricate the PMMA main cover as one piece of body, but just as layer by layer like a usual lithography method. Therefore, the PMMA main cover is patterned as an upper layer and a lower layer separately, and then they are bonded together. The both layers are patterned on 500 um thick PMMA sheets (Goodfellow, Huntingdon, UK) by X-ray irradiation (2.5 GeV, 150 mA) using 15 μm thick Au X-ray masks. Methylmethacrylate (MMA) gel is coated on the bonding surfaces of the both layers of the PMMA main cover and the gas source base. Sequentially, they are contacted, and then pressed with 100 kPa for 12 h.

**Figure 7.**
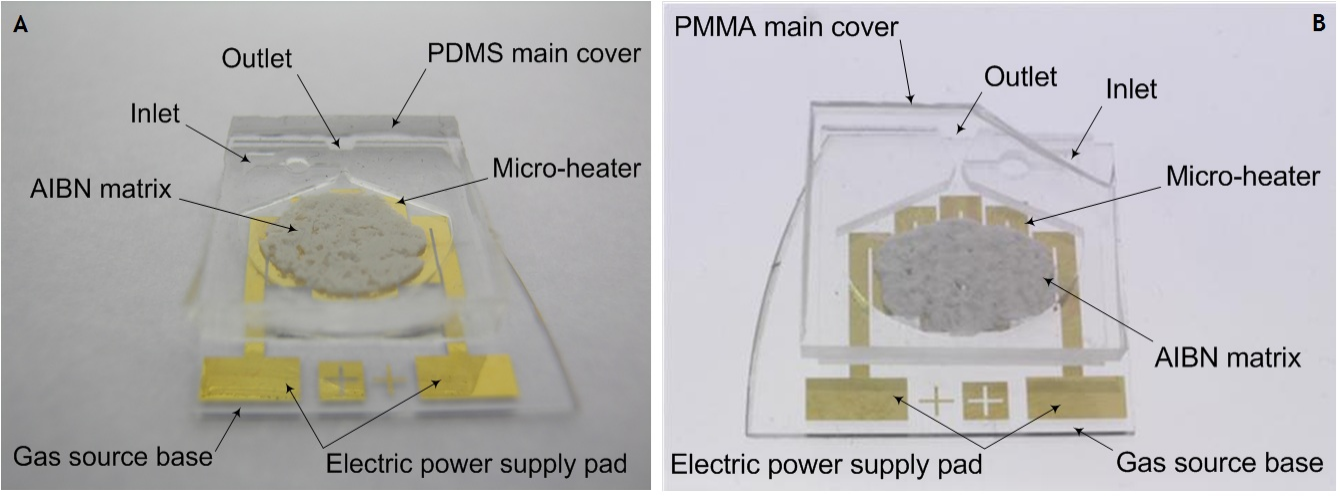
**(a)** The fabrication result of the PDMS gathering tool. **(b)** The fabrication result of the PMMA gathering tool. (Due to transparent property of PDMS and PMMA, the outlines of the fabricated tools are not clearly distinguished.)

Both types of fabricated gathering tools have all the parts in a cylindrical shape with a diameter of 17 mm and a height of 2.3 mm in order to integrate them into a developing versatile capsular endoscope without any scaledown, as depicted in Figure 7. In this research, all the gathering tools are fabricated with a marginal handling part for evaluation experiments. In order to supply an external electric power to the micro-heater, the electric power supply pad is designed and fabricated. When the gathering tool is integrated, the micro-heater will be connected to a power controller in the capsular endoscope, instead of the electric power supply pad.

## 4. Evaluation Experiments

### 4.1. Bonding Force Evaluation

The fabricated gathering tools are under considerably larger pressure than general microfluidic devices, because the nitrogen gas decomposed from the AIBN compound passes the abrupt narrow channel, the vena contracta section. This high pressure can break the gathering tools, so the bonding force must be evaluated.

The bonding force is measured using a bond tester (DAGE 4000, Dage, Aylesbury, UK). The main cover is adhered with a puller of the bond tester using epoxy adhesive (Hardex, Kuantan, Malaysia), and the gas source base is fixed with a jig of the bond tester. The epoxy adhesive which is adhered on the PDMS gathering tool is detached from the PDMS main cover at 24.4 N. Until the epoxy adhesive is detached, the gas source base and the PDMS main cover are not separated. This means that the bonding force of the PDMS gathering tool is more than 24.4 N. The PMMA gathering tool is separated into the PMMA main cover and the gas source base at 9.6 N.

### 4.2. Negative Pressure Measurement of the Inlet

**Figure 8.**
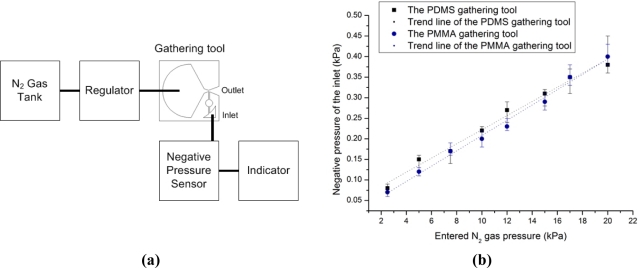
**(a)** The conceptual schematic of a negative pressure measurement setup. **(b)** The graph of the generated negative pressure. The R-squared values of the PDMS and PMMA gathering tool are 0.986 and 0.991 respectively.

The negative pressure in the inlet is measured with respect to N_2_ gas pressure entering the vena contracta section of the Venturi tube. Figure 8(a) shows a conceptual schematic of a negative pressure measurement setup. In order to control the entered N_2_ gas pressure, the AIBN chamber without the AIBN matrix is punctured, and then a N_2_ gas tank is connected to the AIBN chamber. The entered N_2_ gas pressure is gauged with a regulator, which is connected between the N_2_ gas tank and the AIBN chamber. The generated negative pressure is measured with a negative pressure sensor (PSHK-760HAAG, SETech, Daegu, Korea).

Figure 8(b) represents the generated negative pressure. Both types of gathering tools have a similar tendency which is linearly proportional to the entered N_2_ gas pressure. One third of the entered N_2_ gas pressure value is generated as the negative pressure value.

### 4.3. Gathering Experiment with Respect to Contact Angle of the Target Fluid

**Figure 9.**
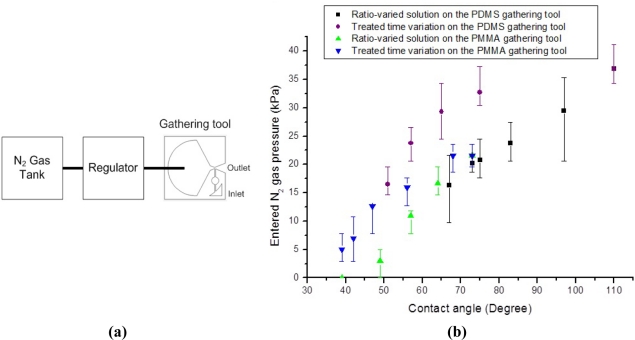
**(a)** The conceptual schematic of the minimum N_2_ gas pressure measurement setup. **(b)** The graph of the minimum N_2_ gas pressure.

The minimum N_2_ gas pressure to start inhaling the target fluid is measured with respect to the contact angle of the target fluid. Figure 9(a) shows a conceptual schematic of a minimum N_2_ gas pressure measurement setup. Like the negative pressure measurement setup, a N_2_ gas tank is connected to the AIBN chamber. The entered N_2_ gas pressure is gauged, when the target fluids, which have different contact angles each other, start to be inhaled.

The surface tension between the target fluid and the inlet is modified by two methods. One is the ratio change between deionized water and acetone. The other is a corona treatment around the inlet. The contact angle around the inlet cannot be directly measured, because of the material heterogeneity and the curvatures around the inlet. In order to compare the surface tensions around the inlets of the PDMS and PMMA gathering tools, contact angles on homogeneous and flat pieces of PDMS and PMMA are measured. Table 1 represents the contact angle with respect to the ratio between deionized water and acetone. The deionized water without acetone has a contact angle of 110° on the PDMS surface and a contact angle of 73° on the PMMA surface. Comparing the results of various fluid ratios in Table 1, the PDMS surface has higher contact angle than the PMMA surface. Table 2 shows the relation between the corona treating time and the contact angle. By corona treating for 10 s, the contact angle of deionized water without any acetone is 75° on the PDMS surface and 57° on the PMMA surface. Comparing the results by different treatment times in Table 2, the PDMS surface has a higher contact angle than the PMMA surface. Generally, as the corona treating time increases, the contact angle between deionized water and treated surface decreases.

Figure 9(b) represents the minimum N_2_ gas pressure. Please note that the X axis of Figure 9(b) indicates the contact angles measured on homogeneous and flat pieces of PDMS and PMMA, not around the inlet. Therefore, at the same contact angle the required minimum pressure can be different. As the contact angle decreases, the minimum N_2_ gas pressure required to inhale the target fluid decreases. In addition, the required pressure to inhale the target fluid is inversely proportional to the acetone ratio and the corona treatment time. In the case of a 6:4 ratio fluid between deionized water and acetone, this mixed fluid has a contact angle of 39° on the PMMA surface. As soon as this 6:4 ratio fluid is dripped to the inlet of the PMMA gathering tool, the PMMA gathering tool inhales this 6:4 ratio fluid without any negative pressure. The corona treating around the inlet of the PMMA gathering tool for over 1 min causes the PMMA gathering tool to inhale deionized water without any negative pressure, as well. These phenomena are suspected to be capillary phenomena.

### 4.4. Gathering Experiment with AIBN Matrix

**Figure 10.**
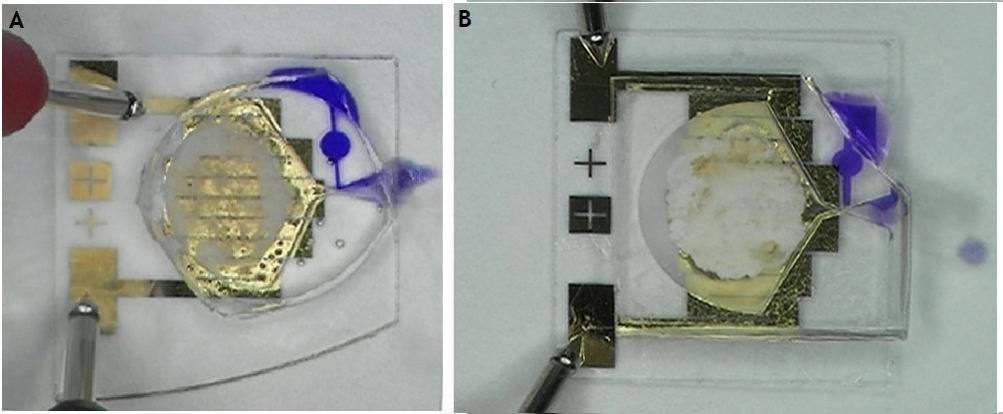
The gathering evaluation experiments. **(a)** The PDMS gathering tool gathers the diluted ink in the reservoir. **(b)** The PMMA gathering tool gathers the diluted ink in the reservoir.

The actual gathering capability of the AIBN matrix is evaluated with diluted blue ink using both types of gathering tools, as shown in Figure 10. The diluted blue ink (78.1° on the PDMS surface and 71.4° on the PMMA surface) is dripped around the inlet using a pipette. Then, electric power is supplied to the micro-heater. Both types of gathering tool have analogous performance. They take approximately 11 s on average to start inhaling, once electric power is supplied. The diluted blue ink takes under 1 s to fill out the reservoir, after the diluted ink starts to be inhaled. The gathered volume is approximately 1.57 μL. The used AIBN compound volume is approximately 25.4 μL. The power consumption is 4.8 W (12 V × 400 mA). The contained fluid does not leak out of the reservoir, until it is dried out. It is attributed to the strong capillary force. The dried-out time of the diluted blue ink is approximately 3 h.

## 5. Discussion

The fabricated gathering tool presents a novel concept of inhaling and containing micro-fluid for a versatile capsular endoscope application. Due to the Venturi tube structure, inhaling mechanism and device structure are simple and reliable comparing to previously reported micro-pumps. Solid chemical propellant replaces a compressed gas tank, which decreases device size enough to integrate it with a versatile developmental capsular endoscope.

The contact angles of gastrointestinal juice (stomach juice) of a rabbit are measured to be 87.1° and 48.2° on PDMS and PMMA pieces, respectively. Those of the tested diluted blue ink are measured 78.1° and 71.4°. Considering these measured contact angles and the required minimum gas pressure [Figure 9(b)], the fabricated gathering tool is capable of inhaling rabbit stomach juice.

The PDMS gathering tool and the PMMA gathering tool show the analogous performance in the gathering capability evaluation using the AIBN matrix ignition. Before the AIBN compound is heat-decomposed into N_2_ gas and by-products, the gathering tool cannot start to inhale. This means that the time to start inhaling is decided by the heating time of the micro-heater to the AIBN decomposition temperature, not by the property of the main cover material. When the micro-heater heats up to the decomposition temperature of the AIBN compound, it seems that most of the AIBN in the AIBN matrix is decomposed instantly. The reason of this instant and full decomposition is speculated to be due to the fact that the AIBN matrix is screen-printed on the micro-heater thinly and widely. This means that the decomposed N_2_ gas generates negative pressure over the minimum negative pressure of the PDMS gathering tool as well as that of the PMMA gathering tool, instantly. The heating time of the micro-heater and the instant and full decomposition of the AIBN compound make the analogous performance of the both types of gathering tool.

During the AIBN ignition experiments, the PDMS gathering tool and the PMMA gathering tool are not broken. Considering the lower bonding force (the bonding force of the PMMA gathering tool), pressure of the generated nitrogen gas in the AIBN chamber is predicted to be under approximately 50 kPa.

The temperature of the back side of the glass substrate is difficult to measure at the instant of the reaction. According to another group’s previous report, the opposite side temperature of the micro-heater for the AIBN ignition is about 55 °C [11]. This level of heat for a short duration does not seriously harm organs or components. Furthermore, the heat generation happens inside the capsular endoscope, and the possible heat damage to organs outside the capsule should not be an issue.

Energy of a capsular endoscope is usually supplied by an embedded battery. A radio frequency (RF) energy transfer function for a capsular endoscope is still under development. This means that all the components of a capsular endoscope are persistently required to decrease their energy consumption until the RF energy transfer function is developed. Even though the 16 mWh (4.8 W × 12 s) energy consumption of the fabricated tool is at the acceptable level, the instant power consumption of 4.8 W is too high compared to other components. This power consumption is dominantly caused by the heating required to decompose the AIBN. Therefore, some other propulsion material which has lower decomposition temperature is required for decrease of the energy consumption as well to minimize any thermal damage.

In order to complete the mission to take a gastrointestinal juice sample out of human body with minimal invasiveness, a capsular endoscope should approach the searched juice in the gastrointestinal tracts, and hold its position during the gathering procedure. These navigational functions are currently under development [16]. Therefore, it takes time to integrate the fabricated gathering tool into a practical capsular endoscope for a gastrointestinal juice sampling function.

## 6. Conclusion

We have fabricated and evaluated a micro-fluid gathering tool for the gastrointestinal juice sampling function of a versatile capsular endoscope. The Venturi tube principal and the use of AIBN enable the fabricated tools to be simple and reliable. In a performance comparison, the PDMS gathering tool can withstand a stronger pulling force, and the PMMA gathering tool requires less negative pressure for inhaling the same target fluid. Due to the instant and full decomposition of the AIBN, both types of gathering tool have analogous performance in the gathering capability evaluation using the AIBN matrix ignition. The pumping capabilities of the fabricated tools show the feasibility for an efficient micro-pump for a lab-on-a-chip as well as a gathering tool of a versatile capsular endoscope. This gathering tool can also be further optimized by modifying parameters such as the micro-heater design, main cover material, and propellant material.

## Figures and Tables

**Table 1.** The contact angle with respect to the ratio between deionized water and acetone.

**Ratio between deionized water and acetone**	**Contact angle on the PDMS surface**	**Contact angle on the PMMA surface**
10:0	110°	73°
9:1	97°	64°
8:2	83°	57°
7:3	75°	49°
6:4	73°	39°
5:5	67°	30°

**Table 2.** The relation between the corona treating time and the contact angle.

**Corona treating time (s)**	**Contact angle on the PDMS surface**	**Contact angle on the PMMA surface**
0	110°	73°
10	75°	57°
20	65°	47°
30	60°	42°
40	53°	40°
50	47°	38°
